# Perceptions of maternity care-seeking and care-giving experiences during a health-system shock: a qualitative study with healthcare professionals and policymakers in the UK, with a focus on care for marginalised groups

**DOI:** 10.1136/bmjph-2025-004361

**Published:** 2026-06-11

**Authors:** Lara Rowell, Tisha Dasgupta, Harriet Boulding, Abigail Easter, Gillian Horgan, Hiten D Mistry, Neelam Heera-Shergill, Aricca D Van Citters, Eugene C Nelson, Peter von Dadelszen, The RESILIENT Study Group, Laura A Magee, Laura A Magee, Sergio A Silverio

**Affiliations:** 1 GKT School of Medical Education, King’s College London, London, UK; 2 Department of Women & Children’s Health, King’s College London, London, UK; 3 King’s Policy Institute, King’s College London, London, UK; 4 Department of Population Health Sciences, University of Leicester, Leicester, UK; 5 Cysters, Birmingham, UK; 6 The Dartmouth Institute for Health Policy & Clinical Practice, Dartmouth College, Hanover, New Hampshire, USA; 7 Department of Psychology, University of Liverpool, Liverpool, UK

**Keywords:** Health Personnel, SARS-CoV-2, Qualitative Research, Pandemics, Obstetrics

## Abstract

**Background:**

Healthcare professionals (HCPs) and policymakers had to reorganise and adapt to maternity services rapidly during the pandemic, and as a result, women’s care-seeking and experience within maternity services were affected.

**Aims:**

This study aims to explore HCPs’ and policymakers’ perspectives of maternity care-seeking and experiences, with a particular focus on marginalised groups. We take a future-focused approach to informing policy to rebuild UK maternity services which remain beleaguered.

**Method:**

Semi-structured in-depth interviews were undertaken with 21 HCPs and 20 policymakers across the four nations of the UK to discuss their perception of maternity care-seeking and experiences during the pandemic. Data were analysed using Template Analysis, based on an extended version of the Candidacy framework.

**Results:**

HCPs struggled to navigate reconfiguration of services and implement changes. It was challenging to strike a balance between the need to protect women from the virus while maintaining high-quality care. The transition to virtual care hindered HCPs’ ability to identify patients’ claim to candidacy, affecting the ability to deliver safe and high-quality care. The standardisation of self-monitoring and virtual care, although beneficial for some, adversely affected those with medical and social complexity. Policy had failed to address inequalities prior to service reconfiguration, making it harder for HCPs to engage with marginalised groups. This included service users with social complexity: lack of social support, mental health problems or belonging to a minority group relating to sexual orientation or gender identity; or medical complexity: those who had to perform self-monitoring of symptoms during pregnancy for any complication, including hypertension, gestational diabetes, additional scans for predisposition to genetic complications, or previous pregnancy loss. A lack of ethnic diversity within leadership roles meant guidelines failed to consider the needs of minority groups who subsequently received substandard maternity care.

**Conclusions:**

This study affirms the negative effects the pandemic had on maternity care-seeking and experience, while highlighting the need for policy to prioritise marginalised groups, patient-centredness and the well-being of HCPs and policymakers to rebuild a resilient maternity service.

**WHAT IS ALREADY KNOWN ON THIS TOPIC**

**WHAT THIS STUDY ADDS**

- This study adds in-depth insights from healthcare providers and policymakers across the UK, highlighting how service reconfigurations undermined safe, equitable care, particularly for marginalised groups and how lack of diversity in leadership contributed to systemic blind spots. This included service users with social complexity: lack of social support, mental health problems or belonging to a minority group relating to sexual orientation or gender identity; or medical complexity: those who had to perform self-monitoring of symptoms during pregnancy for any complication, including hypertension, gestational diabetes, additional scans for predisposition to genetic complications or previous pregnancy loss.

**HOW THIS STUDY MIGHT AFFECT RESEARCH, PRACTICE OR POLICY**

- These findings suggest that rebuilding maternity services will require policies that explicitly address inequality, centre patient and staff well-being, and ensure diverse representation in leadership, with potential implications for how future crises are managed across health services.

## Introduction

 In the UK, maternity care is a public health service characterised by antenatal appointments whereby healthcare professionals (HCPs) support women’s physiological, psychological, and social needs throughout pregnancy.^1 2^ Whilst care remains ‘free at the point of delivery’,^3^ engagement with services remains diminished among women with psychosocial disadvantages such as lower socio-economic status, lack of continuity of care, and reduced social support.^4 5^ Poor engagement with maternity services is linked to adverse outcomes, including preterm birth and perinatal mortality, highlighting the need for accessible, high-quality maternity care.^6^ The onset of the pandemic in 2020, necessitated rapid restructuring of maternity services to mitigate viral transmission,^7^,^9^ including delayed invitation to the labour ward and reduction of partner presence on labour and postnatal wards^10^; rapid ward changes, staffing and service delivery^11^; and tiered escalation of postnatal care from telephone to videocalls to face-to-face care.^12^ These changes placed significant strain on HCPs, who faced staff shortages, burnout and difficulty upholding high-quality care.^13 14^ Consequently, women’s experiences with services were met with dissatisfaction and associated with poorer health outcomes.^15^ It is therefore critical to address the policy and care delivery which shaped maternity care seeking and experience during the pandemic.

This study uses an ‘extended Candidacy framework’ to explore HCPs’ and policymakers’ perceptions of women’s maternity care-seeking and care experiences during the pandemic. The Candidacy framework was initially developed to explore the influences on access to healthcare among vulnerable groups.^16^ We have used the seven constructs: identification, navigation, permeability of services, appearances at health services, adjudications, offers and resistance, and local production of candidacy; with the addition of two constructs: hostile bureaucracy and intercultural dissonance, derived from a systematic review on factors affecting maternity care-seeking in high-income countries.^17^ Where studies have focused on the changes, utilisation and access to maternity services during the pandemic, the synthetic construct of Candidacy provides a framework from which the interaction between social and medical complexities on an individual-, health service-, and joint-level influence access to healthcare.^18^ With the use of the Candidacy framework, we aim to explore the inequalities exacerbated by the pandemic and highlight the pressures faced by HCPs to develop a more resilient maternity care system.^19 20^

## Methods

Full details of the qualitative methodologies used in the RESILIENT study have been published elsewhere.^18^

To summarise, women, partners, HCPs, and policymakers were invited to semistructured in-depth interviews (IDIs; May 2022–February 2023; see online supplemental appendix 1 for Interview Schedule); using a purposive sampling recruitment strategy. This paper presents data related to HCPs and policymakers; interviews with women and partners have been analysed and published separately.^21^ Those who had been involved with policy relating to NHS maternity care or those who themselves had delivered maternity care in the NHS during the pandemic were eligible. Sampling criteria were used to achieve diversity in ethnicity, geographic area across the UK, as well as local, regional, and national foci for policymakers. We sought information on participants’ lived experiences during the pandemic health-system shock, specifically: providing maternity care, their perceptions of those seeking care, their view of NHS maternity services during the pandemic, and their perspective on the availability of information, changing policies, and adaptations to rapidly changing guidelines.

Semi-structured IDIs (N=41) were conducted with HCPs (n=21) and policymakers (n=20). Sample size was based on the approximation of twenty participants required per stratification in thematic framework analysis which was used for the other analyses emanating from The RESILIENT Study.^18^ Table 1 presents participant characteristics. Participants came from a range of professional backgrounds including Midwifery, Obstetrics, Neonatology, Health Visiting, Paediatric nursing, Policy and the Civil Service, and Non-clinical research; with the 2–5 years in their current role being most commonly endorsed (39%). Most participants self-identified as White or White British (83%).

**Table 1:** Participant characteristics

Geographic region			
South London	9	0	9
London Other	3	0	3
East of England	2	0	2
Midlands	2	0	2
North East	1	0	1
North West	0	0	0
South East	0	0	0
South West	1	0	1
Wales	1	0	1
Scotland	1	0	1
Northern Ireland	1	0	1
National reach (policymakers)*	0	14	14
Regional reach (policymakers)*	0	5	15
Local reach (policymakers)*	0	1	1
Ethnicity			
White–English/Welsh/Scottish/Northern Irish/British	12	13	25
White–Irish	1	1	2
White–Any other White background	4	3	7
Asian/Asian British–Any other Asian background	0	1	1
Asian/Asian British–Bangladeshi	0	0	0
Asian/Asian British–Chinese	0	0	0
Asian/Asian British–Indian	0	0	0
Black/Black British–African	0	0	0
Black/Black British–Any other Black/African/Caribbean background	1	0	1
Black/Black British–Caribbean	0	1	1
Mixed–Any other mixed/multiple ethnic background	1	0	1
Mixed–White and Asian	0	0	0
Mixed–White and Black African	0	0	0
Mixed–White and Black Caribbean	0	0	0
Other ethnic group–Any other ethnic group	1	0	1
Other ethnic group–Arab	1	0	1
Prefer not to say	0	1	1
Index of Multiple Deprivation			
1 (most deprived)	0	0	0
2	0	0	0
3	1	3	4
4	1	1	2
5	3	1	4
6	2	1	3
7	4	2	6
8	3	0	3
9	0	4	4
10 (least deprived)	2	1	3
Prefer not to say	5	7	12
Age at Interview			
18–25	0	0	0
26–30	1	0	1
31–40	5	1	6
41–50	4	5	9
51–60	8	8	16
61–70	2	3	5
Prefer not to say	1	3	4
Professional background			
Midwifery	5	9	14
Obstetrics	10	5	15
Neonatologist	0	2	1
Nursing/health visiting	2	2	3
Paediatric nursing	1	0	1
Other clinical specialty	2	0	2
Policy/civil service	1	3	4
Non-clinical research	0	1	1
Years in current role			
<2 years	3	6	9
2–5 years	6	10	16
6–10 years	4	1	5
11+ years	8	3	11

* For policymakers, data were collected on the geographic scope of their work, rather than physical residence.

Policymakers most often had a national brief (70%), with others having regional responsibilities (25%) or local portfolios (5%); whilst HCPs were most often from London (57%), with representatives from other regions of the UK (East of England, Midlands, North East, South West; 29%), as well as from the other three nations of the UK: Scotland, Wales and Northern Ireland (each with 5%).

Transcripts were subject to a Template Analysis,^22^ reflecting the extended version of the Candidacy framework,^17^ the initial coding template (table 2). These factors can be clustered as individual-level, health-system-level, or joint-level factors. The ‘extended’ version offers an additional two factors to the Candidacy framework’s original seven.^16^

**Table 2:** Candidacy framework factors

Candidacy framework factor	Individual-level	Health system-level
Existing factors^16^		
Identification	●	–
Navigation	●	●
Permeability	–	●
Appearance at health services	●	–
Adjudication	–	●
Offers and resistance	●	●
Local production of candidacy	●	●
New factors proposed^17^		
Intercultural dissonance	●	–
Hostile bureaucracy	–	●

## Results

Results have been organised according to the extended (7+2) candidacy framework, with the two additional constructs specifically addressing migrant experiences of healthcare; and in the context of this study: specifically HCPs’ and policymakers’ perspectives of women’s experiences of maternity healthcare access, engagement and utilisation. The analytic template is therefore arranged to address each part of the candidacy framework, organised into a tripartite set of themes which discuss individual-level, health service-level and joint-level constructs from within the candidacy framework, as below. Tables3^5^ present exemplary quotations to support each theme.

**Table 3:** Candidacy framework–individual constructs

Identification of Candidacy	Appearances at health services	Intercultural dissonance
*And we also got a suspicious decrease in women turning up with emergency complaints to MAU, which is A&E for pregnant women. So we worried that people were sitting at home having symptoms and not doing anything about them. So we put out quite a lot of information saying, “These are still the things you need to attend with. Don't sit at home because you're worried you'll get COVID.” And then we did overall have a slight increase in the stillbirth risk, either because of COVID, we're not sure, or because people weren’t attending with things they should have been. – H019* *So when these decisions were made, they were not made based on what’s best for women, women having babies, pregnant women, labouring women. And the same applied to the centralisation of home birth services, because I know in certain areas it was a decision made for women by the Service Manager based on availability of staff or availability of ambulances. And I think as anything in healthcare, it’s a matter of describing the risks and benefits, and then it’s an informed choice that women make. - M011* *My perception would be that there was a lot of information available. However, I know from speaking to service users that they felt that there wasn’t enough information available, certainly from a maternity perspective. We did some surveys for our service users, and they definitely felt that they weren’t given enough information, certainly about services and things that were available. – M004* *I think a few things worked really well. So, very quickly, our midwives came up with a whole load of videos that they basically developed for people who’d just had a diagnosis of gestational diabetes, for example. So that was fantastic; they posted them online, so all our patients, all our inpatients, all our outpatients have electronic patient records so that was absolutely amazing, because basically wherever you were, you could look up your data. They could post all this information online, and it was hugely, hugely beneficial. – H011* *So, some women love self-monitoring, and they love the additional responsibility and sense of engagement and ownership and empowerment that it gives them for looking after their health and their baby’s health during their pregnancy. Others find being given a monitor and being asked to take on that responsibility for monitoring their blood pressure somewhat overwhelming and anxiety-producing. So, it’s certainly not a one-size-fits-all intervention. – M002* *I think that’s what this crisis has done; it has taken us backwards and it has put a lot of limitation on women’s choices and women’s agency and autonomy. So I think it’s one of the things that it’s incredibly unbelievable, but how fear and an unprecedented situation can just take us backwards because of other things becoming a priority and fear playing a big role into that. So there is a lot to do, it’s almost like we need to undo what [laughs] this pandemic has done before we can move forward. – M011* *I knew, that women’s health is not a priority for this Government, nor for this society. I think we always overlook the needs of women, and we disregard the public health implications of disregarding women’s health. And it’s something that we absolutely need to be on the watch for all the time because it’s never made a priority, so I think that’s something that we as women, and women in policy, have to always have as a top priority on the agenda, is to make sure there is some spotlight on women’s health and the long-lasting implication of neglecting it. – M011*	*most of them have been able to express themselves and are often more comfortable expressing themselves and being more realistic and able to share much more vital information than they could do physically or in person. So this has boosted morale and participation in clinical trials and coming out to express themselves to healthcare professionals much better than they could ever do in person. So, yes, the women have really been more involved and enthusiastic about the virtual participation. – H002* *They also very crucially told us that it was much harder to develop rapport and trusting relationships with their health professionals when they were only ever hearing them over the telephone or seeing them over a video link. So, they felt it was harder to develop rapport and trust with their health professionals, and harder to raise concerns around their pregnancy, and particularly raising mental health issues or worries. Just one last point, they also said that they found the telephone consultations much… they felt shorter, and they felt more transactional, and they felt that it was harder to raise the additional niggles and worries that they might have more easily been able to arrange if they'd seen their health professionals face to face. – M002* *I would quite often do bookings from home, on [secure NHS video call service*]. *So those were five or six bookings over like this, over a screen. I don’t know who’s in the other room, I don’t know who’s around her, I’m asking personal questions, can anyone hear, does she feel comfortable to talk to me about it, is she really going to disclose about an STI or severe mental illness if her mother-in-law’s around? – H006* *I think what was less good was we know that it’s more difficult to pick up domestic abuse, child abuse, if you’re not visiting people in their homes, if you don’t see them, if you don’t have the kind of personal and in real life contact. So I think for certain groups it was beneficial and it was more convenient and it was more personalised, but for other groups in terms of health inequalities, it has widened I think the health inequalities for women that are less able or in a more vulnerable and high risk situation, so I think it’s very difficult balancing those two elements; I think it needs to be personalised, you cannot standardise the hybrid care. – M011* *I think that the empowerment of women, the known data on the empowerment of women within face-to-face appointments and how staffing groups hear them, there is already an issue with face-to-face appointments and I feel that the virtual appointments just exacerbated those. So I think there were unfortunately…we did what we needed to, but there were several hits in providing virtual appointments which meant that these women were further disadvantaged. - M016*	*There’s still women not having interpreters, when they’re using maternity services; how can they make informed choices? And things like that. So I think it’s look back at the lessons, and do something, rather than, when we have another pandemic, possibly we could do this, that and the other, and then realise, there were issues that were raised this time around, which no one has followed up on. – M014* *And also making sure that any information that is developed is relevant to those various population groups, with an absolute focus on health inequalities and those groups who are seldom heard and perhaps have got additional needs and complexities – H018* *So those respected people within their own local communities, who actually spoke languages that sort of engage with those perhaps seldom-heard individuals, to make sure that information, particularly around vaccination and infection prevention was getting through to the right communities. Faith leaders, again, were incredibly helpful to us. But I think, as with everything, it takes that little bit of time to actually mobilise that level of support and those community influencers as well. But it’s probably one of the significant legacies to the whole Covid 19 pandemic, is the presence of those now community champions who continue to work with the organisation to convey key messages about health-related issues. – H018* *So as we were getting data that some ethnic groups were at higher risk, the fact that we didn’t really have wide diversity in the group, I think guided how we responded to it. So it very much became the problem of ethnic minority staff groups to identify themselves at risk. And I very strongly felt that that wasn’t the right thing. I thought as an organisation that’s got such a diverse workforce, we should have undertaken that accountability and responsibility. Because again, we were relying on people who, through many other studies and data have told us that they don’t feel empowered, don’t feel listened to and feel discriminated against and I think we added to that. – M016* *if some of the racial inequalities that had been identified in maternity services, if they were addressed previously, perhaps the impact would not have been that great. But we’re theorising here, if and but, I don’t know, but I think that there wasn’t a focus until the data showed that there are high numbers of Black women being hospitalised who are pregnant with Covid-19. And then, there was something, but we already know the issues around maternity services, so I think some of this should have been addressed, and perhaps the focus would have already been there to look at this during Covid, rather than suddenly seeing this data and a reaction. – M014*

**Table 4:** Candidacy framework–health service-level constructs

Permeability of services	Adjudication	Hostile bureaucracy
*Because 60% of the caseload at [hospital name] is out of area, it’s a huge amount. So some women were coming all the way from [county*]. *Some women were coming from [county], [county*]. *So, for those women to come in on the train all that way, or if the trains were on strike or cancelled or whatever [laughs], then that worked out well. It worked out well if they were anxious about coming into the hospital, especially because I think everyone sees [hospital name] as this place where all the diseases are, and there’s more people with illness, and all that stuff. So I think they always feel a bit more risky coming into hospital. But I think that’s what worked well, was that there was the option, and easier for women. I didn’t like it. I prefer seeing them in face-to-face, body language, all that stuff. – H006* *But inevitably, it’s the more vulnerable and the less able people in society who have significant problems there, and that’s people who have got more chaotic lifestyles, people who don’t have English as their first language; there’s a whole range of individuals who by definition will not be able to respond to that as well as someone who has got smartphones and knows how to contact people and how to find their way through the minefield of the health service. – M013* *I think it came back to, if you look at the index of multiple deprivation, and the groups of women who also then, ethnicity wise have this intersectionality crossover, so they’re from groups with high deprivation and from ethnic minority groups, the ways in which we were providing virtual appointments was really difficult. Not all of them have phones. They’re about the only group who don’t all have phones and certainly not all had smartphones. We were disseminating a lot of information about the virtual appointments through our digital platforms and they don’t have access to them. They don’t use them. – M016* *And particularly for someone who is pregnant and going into hospital, their partner was the person they want with them and within their bubble, but they’re not getting that support, because at the time, you had to go in on your own. And I can imagine a lot of women are traumatised from that experience, which would impact on any future pregnancies they may have. – M014* *Well, I think patients on the whole found electronic information, so, Facebook pages, updates on NHS websites, reasonably easy to understand and follow. Although recognising that not all groups of women who are going to maternity care services are digitally savvy, digitally confident, and that Trusts and NHS England and the health system should not assume that if they publish everything on electronic platforms that that’s going to reach everybody. So, thinking through being able to deliver information on sources that are going to be accessible to the full range of women going through the services is going to be important. – M002* *So there is something about how accessible the information is produced, how it reaches to communities. So I think there should be more work with local voluntary organisations, possibly local radio, in order to reach those communities. And also, it’s about the trust that people have of Government, certain information, part of this is to do with their own experiences or communities’ experiences of the health services. So I think there could have been a lot more done in terms of information, how it was provided and access to information. – M014*	*But I feel like they didn’t get that kind of relationship that you get when you see someone face-to-face all the time, you know them a bit better. And they might have seen different people, because of sickness, because of short staff; they could have seen different people within the perinatal mental health team every time. And then we’re trying to organise pre-birth planning meetings over a screen as well, so you’re having this, how are you feeling, with all these people on these different screens, you know? Trying to make a plan for your recovery post-birth, and quite often, I was in a shared office, so I’d have my headphones on, so that obviously nobody could hear anything, but the women would see people walking past me, you know? And if the office got really loud, I was like, [laughs], trying to keep it quiet. – H006* *I think maternity is very much a relationship-based profession, but also you can’t palpate someone over video, you can’t take their blood pressure over video. So you can have consultations and discussions over video, but there are elements of care which are routed in the physical contact between the care giver and the person having a baby, so I think it left women feeling very vulnerable and isolated. – M006* *there’s women out there who are challenged with domestic violence situations or ongoing mental health or safeguarding concerns, that there was sort of a blanket approach to ‘we’ll do everything virtually’ so the identification of some of those issues would have been significantly delayed. – M015* *So you're working with families who may not recognise that they have a problem themselves or their baby is in distress, that their child’s got a health condition. So you are trying to sift information. You're reading the situation that you're in, so that’s the context in which families live, the interaction between the mother and baby, all the physical symptoms, you're trying to put pieces of a jigsaw together. And so that had never been tested using virtual methods. – M008* *I guess no more than usual, that is like it’s difficult assessing people on the phone even if they call up and say, ‘Oh I’m bleeding a bi’” and you ask them to describe it, they are like, ‘Oh it’s like kind of reddy colour,’ and then you have to be like, ‘Okay, well is it dark red, is it pink, is it like the size of a 5p?’ Phone calls for any sort of clinical assessment are pretty hard when people are relying on the public to describe something quite accurately for you to then decide whether you need to get them to come in or not. – H013* *But that’s clearly a risk, when you're speaking to somebody on Zoom, you can't see if there’s some abusive partner on the other side of the screen indicating to her, whereas at least if you're in the antenatal clinic and the partner’s there with her, you can watch the body language and you can sense that there’s tension between them, or if she’s afraid of him or whatever. But it’s almost impossible to get that by Zoom or even more so over the telephone. So that’s a big disadvantage. – M018* *I remember especially in London, a lot of the clinical queries we were getting, it was terrified midwives going into labour wards, seeing women that were sick; they felt they didn’t have enough PPE to deal with them. All of those things [sigh] were terrifying, frankly, and we didn’t think the response was good enough to start with. – M011* *You wanted to give care. You did not want to be faffing around with a headset and a microphone and a camera that did not work. You just wanted to see women and give them care. – H001*	*when you look at all our systems of investigation around safety incidents, the lack of interpreters is still an issue. My professional opinion about this is that when we have our resources restricted, as we do financially in workforce, that the first things to go will be the things that we think are least important. And despite what we say about the importance of interpreting services, those are the first ones to go. – M016* *although we say reducing health inequalities and inequities is a priority of the NHS, all the systemic and institutional biases kick in when you have this sort of situation. And so no-one was particularly looking at this particular group of people apart from the fact that oh, ethnic minority groups get Covid more and might do less well. So I think it showed the ingrained views, perceptions, of people who make policy. It is not driven by clinicians or clinicians who work in areas or have an understanding because their core populations are highly diverse. – M016* *But the bias in this case was the language barrier, because a lot of people in the UK have different native languages and most people, or should I say about 20 per cent, 30 per cent of people in the UK don’t fully understand English and are not really fluent with the language. So this is kind of a problem for a lot of people because not all of them have a personal interpreter of some kind of a support person who could explain certain things to them and this is a bias which needs to be checked and evaluated as soon as possible. – H002* *I think there’s also an issue on how some communities who would probably be supported by specific minority ethnic voluntary organisations, how they were going to be supported, because these voluntary organisations were themselves very much reduced pre-pandemic, in terms of funding and what organisations were available. So it’s in terms of getting the support to the communities; those organisations were sort of limited in their resources. – M014* *So for example, the fact that you were telling people to perhaps use different bathrooms, or people should self-isolate; many people from those communities are in overcrowded accommodation. So their ability to do that is restricted, in the same ways as going out and walking in green spaces, we know that these communities are less likely to live in places where they have access to green spaces – M014* *However, there was a real issue with that, because we went to the traditionally appointed formal leadership role, which meant that the first time we all sat face-to-face in the [name] lecture theatre, it was very obvious to me that the diversity of the leadership group was non-existent, apart from me at the time. And one of the community director of ops is also Asian and female. And I thought that was an issue, given my interests and healthcare management system knowledge, so I knew that we had an issue with the diversity. – M016*

**Table 5:** Candidacy framework–joint-level constructs

Navigation	Offers and resistance	Operating conditions and the local production of candidacy
*But that was the most difficult thing, was an anxiety that women weren’t getting quite the quality of care that you would have hoped, potentially because they were being missed. So instead of having a phone call every three weeks or four weeks. And there is a bit of a blip in the GDm-Health system in that we, in [hospital name], we have many women who haven’t been archived. And so there’s such a big list of women…we have big lists of women anyway, but it was not as easy to try and identify those women who were not getting called or who weren’t checking their sugars. Whereas previously, if they didn’t pitch up to clinic, you knew that they hadn’t pitched up to clinic. – H015* *women told us that they often found that the organisation of the remote consultations was confusing and not clear, and they were often having to wait around quite a long time for a phone call or a video consultation. They found that they felt more isolated from their health professionals and from other would-be mothers-to-be, so the remote care amplified the experience of being isolated in their pregnancy, which of course was amplified by the lockdowns that were forced on them by the pandemic as well. – M002* *If you shut families down and you lock them down, then vulnerability goes up. And that’s the role of the health visitor, is to work alongside families, to spot vulnerability, and to support families early, to stop small problems from becoming big ones, for a whole raft of health and social care reasons. – M008* *because we didn’t want people coming to hospital. They were left on their own, and even if they had to come as an emergency, their partners were not allowed to be there, just to hold their hands, during a very difficult time of miscarriage. And there are lots of horrific stories where people miscarried in the toilet, having a foetus in their hands on their own, or in a ward. So I think the provision of care for miscarriages was absolutely shambolic and awful, and well below par. – H007* *And the other element that’s become very clear throughout the pandemic is the importance of peer networks and peer supports, and that social infrastructure that often women lost and the impact that that actually has. And the other element was bonding in terms maternal mental health that we were particularly concerned about and those elements such as speech and language as well. – H018* *I think since the beginning, we knew from looking at, well, common sense but also looking at some of the evidence, is that if you strengthen community based care, you’re actually breaking the chains of transmission, you are stopping people from being centralised in a way where it’s easier for them to catch the virus and spread the virus and all of that. And I think if we had done that from the start, we could have scaled up some of the community based services and just relieved some of the pressure on the hospitals and the ambulances and all of that. – M008* *So, some areas delivered really great services and other areas literally it felt like they shut shop, they offered so little to families, for all sorts of different reasons. Maybe they were redeployed, or they already had a workforce shortage that was significant. And so too many vulnerable families have been left without the support that they needed. And actually, that matters because of the impact that that will have across both the parent and the child’s life course. – M008*	*declining having a blood transfusion even if she had a serious haemorrhage and was willing to die because she didn't want to receive blood from somebody who might have had a COVID vaccine. That’s how far it had gone. So, the disinformation out there, which is really hard to counter, especially if you're at all hesitant. So, the problem is the disinformation gets hold of members of the community. And then unless you've been completely proactive and you sort of see that off at the pass, then it will become embedded into people’s minds. And once somebody’s got a perception, it’s really hard to shift it. – H004* *People who were frightened of vaccines were very anti-vaccines, people who didn’t like hospitals and who were frightened of hospitals, this all fed into that, so it all just amplified a lot of the fears and difficulties that people had really. – M013* *If somebody refused a COVID swab on the way in, then they were treated as COVID positive, so if their baby became unwell, then their baby had to be isolated and all of that was to keep other people safe, but it was also… how that impacted on mothers was, ‘Well, you are treating me differently because of the choices I have made’ and people should not be treated differently. – H003* *People were worried about their own safety in terms of being in hospital and treating patients with Covid. – H007* *Some women, I think, stayed at home because they were watching the news every night and the news was full of hospitals, full of dying people, etc., etc., and people wearing hazmat suits, and they thought, “I really don't want to go into this hospital and risk getting infection, or going on the bus and risk getting infection.” And also, altruistically, they were seeing hospitals, doctors, nurses, midwives are under huge pressure, and altruistically, they were saying, “Well, I don't want to add to the pressure of these poor people looking after very sick patients.” – M018* *the second thing was there were so many messages went out about protecting the NHS, only using them if you absolutely had to, that a lot of people didn’t want to trouble you, so they thought it’s alright, it’ll be fine, I’m not going to worry people, I don’t want to go in and have gone in unnecessarily’ and so we did a lot of messaging that if you are worried, phone us, and we can make an assessment whether we think it is a worrying thing or not. But I know there were a lot of people said, “Well we didn’t come in because we knew how busy you are”. – M019* *And I know from experience talking to people where there’s a clear indication, a clinical indication that some medical intervention is required in terms of starting some medication and some of the women can be quite resistant to that, and that can happen when you're in-person, but actually, it’s easier for the woman to, I think, in a virtual consultation, for the women, because I'm going into their space, it’s easier for them to say, “Well, actually, no, I don't want that now,” or, “I want to defer it,” or, “I can make the changes.” Now, you know, there was always a situation where people might have wanted to defer starting medication or were a little bit more resistant or felt that it could make changes. I've just noticed it’s more pronounced in virtual consultations. – H020*	*I think pregnant women, especially at the very beginning, viewed Covid as really scary and something that would really affect them and their baby, and I think that then meant that people were less likely to come into hospital when they needed to and a massive increase in people like wanting home births and that sort of thing. So I think there was definitely this kind of idea of hospitals are bad and you want to stay away from them. So I think that was definitely a case. – H021* *And we probably underestimated the power of fake news, feeding on people’s fears and people’s vulnerability to conspiracy theories and things like that. – M018* *I think that’s difficult. I think, you know, journalists want to sell their papers. They don't always sell the truth. Well, they always say the truth, but they wrap it up in their own version of it. And for a very scared, nervous public, that was difficult. I think the media didn't help to manage the message well and it caused huge problems for the NHS, and for organisations like us. – M007* *So, the dissemination of information that people were confident about through official channels felt good. There was, of course, further dissemination of information through social media, some of which was good quality information and some of which was pretty poor quality information. It was then a really complex challenge to counter some of the disinformation that was going through those channels. And I think that some of those harder-to-reach groups were better plugged into the social media side of things, and were therefore, potentially exposed to more of the disinformation than the official information that came through perhaps less, sort of, socially based communication channels rather than the official communication channels. – M017* *In terms of my view of the government messaging, I think this government is awful, so I think their messaging was pretty atrocious. And this wasn’t really the government’s… I guess it was NHS England’s kind of fault but the whole messaging about the vaccine with pregnant women I thought was like we’ve really caused them a disservice. So I think overall it was a bit chaotic, but I think it definitely did give the view to women that this was something really scary and they were really anxious. – H021* *I hope that we learnt the impact of public messages. You know that initial wave and the initial lockdown - don’t come to hospital, stay at home – I feel that clearly had a tremendous effect, not just on pregnant women but also on other patients and we’ve seen that. – H005* *I think there were decisions made for women without women during the pandemic – H003* *And it was absolutely imperative to be there and make sure that maternity was on the agenda. Because there were certainly people in the organisation initially who thought, well, this is a thing that’s going to affect old people, almost like, well pregnant women won’t get Covid. There wasn’t the thought that it was the whole population problem. So making sure that the needs of maternity services were kept on the agenda. So we have a service that can’t stop running, that isn’t always understood by the rest of the hospital. In the same way as A&E can’t stop running. We keep running. – M005* *started going to see a psychologist about it because I felt that people were losing sight of the job they were supposed to be doing. It became about COVID and not about the work that you were supposed to be doing, which was protecting women and babies and giving them options and caring for them in labour and placing them first. – H003*

1. Individual-level factors:
  - ‘Identification of candidacy’ (self-acknowledgement of the necessity of medical attention for symptoms);
  - ‘Appearances at health services’ (individuals’ ability to assert their candidacy);
  - ‘Intercultural dissonance’^+1^ (experiences of distinct differences in social and medical norms and culture).
2. Health system-level factors:
  - ‘Permeability of services’ (ease with which people can use services);
  - ‘Adjudication’ (HCPs’ judgements dictating progression of individuals’ candidacy);
  - ‘Hostile bureaucracy’^+2^ (discriminatory policies and precarious administrative practices).
3. Joint-level factors:
  - ‘Navigation’ (learning about and negotiating services);
  - ‘Offers and resistance’ (declining to accept medications or referrals);
  - ‘Operating conditions and the local production of candidacy’ (local factors influencing candidacy, including availability of resources and patient–HCP relationships).

### Individual-level constructs

#### Identification of candidacy

The decision to characterise pregnancy and postpartum women as ‘vulnerable’ affected their ability to identify candidacy and seek care. HCPs reported that women were hesitant to acknowledge their symptoms and attend hospital visits. Moreover, policymakers reported that the ‘authoritarian’ approach to implementing COVID-19 guidelines lacked patient centredness. Women felt this absence of shared decision making and struggled to seek the care which best-suited their needs. The scarcity of information available about COVID-19 and its effect on pregnant women hindered HCPs’ ability to pass on information to patients. Subsequently, women lacked the appropriate knowledge to guide decisions about their own health and were uncertain in their ability to assess their need to seek care. However, in some rare cases, HCPs used novel methods to quickly translate official guidance into information for women, via WhatsApp groups for example.

In many cases, self-monitoring enhanced women’s ability to identify candidacy; however, the standardisation of self-monitoring disadvantaged women who were less comfortable with the technology and/or less able to identify symptoms warranting care. Moreover, a large proportion of women reported feeling unsupported by the healthcare system throughout the pandemic, an issue which was exacerbated by exclusion of partners from antenatal and labour care, which left women with the responsibility of identify candidacy for themselves and their baby without the support of their family.

Policymakers conveyed their concern over how the restrictions to maternity services were implemented. They stated that these decisions were made under a bureaucracy which did not prioritise women’s health. As a result, women’s reproductive choices were limited whereby they were no longer able to dictate where they wanted to give birth or access necessary reproductive care.

#### Appearances at health services

Appearances at health services were characterised by women’s ability to express their concerns within maternity services. This ability was challenged by the rapid reconfiguration of services whereby women no longer had the same lines of communication with HCPs as they did before the pandemic. For example, some women felt more confident discussing care through virtual consultation; however, others found this hindered their ability to build trusting relationships with HCPs. At times, consultations were shorter, less frequent, and impersonal. HCPs perceived that this affected women’s ability to disclose sensitive and essential information about their pregnancy. HCPs acknowledged that this widened existing health inequalities for harder-to-reach communities, who for example, are technologically disadvantaged.

#### Intercultural dissonance^+1^

Efforts to engage with harder-to-reach communities varied among UK Trusts (organisational units within the NHS who provide aspects of healthcare i.e. a group of hospitals may fall under one singular ‘Trust’). Some Trusts were able to engage with faith leaders or valued members of the community to better-understand the needs of their community. However, in some cases HCPs conveyed their concern over groups who became disengaged with health services due to service reconfiguration and a lack of will to engage with these communities in the midst of the pandemic. Policymakers suggested that had these inequalities been addressed prior to or throughout the pandemic, these minority ethnic groups would not have been so disproportionately affected by the pandemic.

### Health Service-level constructs

#### Permeability of services

The impact of the pandemic on permeability of services was mixed. On the one hand, the transition to virtual care appointments increased accessibility to services for women who saw travelling to hospital as a significant burden, financial or otherwise. On the other hand, virtual care limited access to services for groups that were technologically disadvantaged or faced a language barrier. Moreover, maternity services were not accessible to partners during the pandemic, which left women to face challenging situations alone. While HCPs and policymakers used a variety of online platforms, including social media, to make health information more accessible to the majority throughout the pandemic, they recognised that more work was required to make information accessible to local communities, who do not engage with mainstream media.

#### Adjudications

HCPs’ ability to assess patients’ claim to candidacy is characterised by the relationship they built between themselves and their patient. HCPs reported that the transition to virtual care impeded their ability to create such a trusting relationship, and to identify non-verbal cues, safeguarding issues, and patients’ need for further support. A lack of PPE and working technical equipment also affected HCPs’ ability to deliver safe and good quality care—a problem which emerged early on in the pandemic.

#### Hostile bureaucracy^+2^

HCPs described their difficulties in providing interpreters during virtual consultations, disadvantaging women from ethnic backgrounds. Policymakers were concerned about the lack of ethnic diversity within leadership roles, which they perceived as resulting in the under-representation of the needs of minority groups. Despite living in a multicultural society, policymakers felt there was a lack of will to address the existing racial inequalities within maternity services, which led to substandard care.

### Joint-level constructs

#### Navigation

HCPs reported concerns over the reconfiguration of maternity services as they felt that women were not receiving the appropriate standard of care. HCPs felt that the choices behind the reconfiguration of maternity services did not consider the negative impacts it would have on women and children. For example, the decision to stop antenatal classes and home birthing services left women feeling isolated and unsupported. Restricting access to these services highlighted the importance women place on peer support and social connections during pregnancy. In an attempt to control infection rates, maternity care was largely centralised to hospitals which meant that community services, such as deployment of health visitors, were stopped. This raised concerns over women’s safety as the detection of safeguarding issues and assessment of the home environment had to be undertaken virtually. The reconfiguration and thus standard of services varied significantly between Trusts within the UK, which led women to seek care in specific hospitals or geographic areas where they would feel better supported.

#### Offers and resistance

HCPs perceived that women’s personal beliefs and judgements about the COVID vaccine characterised their willingness to accept it. The uncontrollable spread of misinformation was noted as affecting vaccination uptake among the perinatal women. HCPs reported that women were fearful of exposing themselves to environments wherein they would be in contact with COVID which resulted in a decline in the number of women seeking care in hospital. This was largely influenced by the circulation of information through news outlets and the media which influenced women’s decisions to seek care. Additionally, some HCPs reported that patients were more assertive in declining medical care offered when this was done by virtual consultation, compared with in face-to-face appointments.

#### Operating conditions and the local production of candidacy

The dissemination of information online heightened women’s fears of COVID, increasing their levels of stress and anxiety. As a result, women’s engagement with social media had a significant impact on the way they received and acted on information about the pandemic. Further, mixed public messaging on COVID-19 guidelines from the government heightened women’s anxiety and uncertainty during pregnancy. HCPs and policymakers felt that women were not a priority on government agenda and that decisions being made about maternity care were often politically charged. Moreover, the immense pressure felt by HCPs to accommodate high standards of maternity care, while still complying with stringent infection control guidelines, affected their psychological well-being, and ability to care for women.

## Discussion

### Main findings

This study explored the perspectives of HCPs and policymakers involved in maternity care during the pandemic, revealing insights into the challenges faced and highlighting the need for health service adaptation to support a more resilient maternity workforce.

The pandemic necessitated the significant restructuring of services however, often at the detriment of women’s care-seeking and experience. HCPs struggled to deliver high-quality care amidst rapidly changing conditions, while policymakers struggled to uphold ethical and respectful care under stringent infection control measures. A key challenge was the lack of clear, consistent information, which made it difficult for HCPs to guide women through the already daunting journey of pregnancy. Frequent updates to guidelines and poor dissemination of information resulted in women turning to informal sources such as friends, social media and news outlets, which perpetuated misinformation and care refusal.

The abrupt shift to virtual care disrupted HCPs’ ability to communicate with, build relationships with, and make judgements about women’s need for further care. Women’s experience with virtual care was ambivalent; for those who were medically or socially disadvantaged, it was perceived negatively, whilst yielding positive responses from women who found attending the hospital logistically demanding. Reduced access to in-person appointments, limited birth partner involvement and birth options were perceived unethically by all.

Moreover, our participants agreed that marginalised groups struggled disproportionately to identify candidacy and seek care due to changes to service provision made throughout the pandemic. This included service users with social complexity: lack of social support, mental health problems, or belonging to a minority group relating to sexual orientation or gender identity; or medical complexity: those who had to perform self-monitoring of symptoms during pregnancy for any complication, including hypertension, gestational diabetes, additional scans for predisposition to genetic complications, or previous pregnancy loss. When face-to-face contacts with HCPs were reduced, services became inaccessible and harder to navigate. Women who struggled to adapt to these changes became further disengaged from maternity services and HCPs struggled to reach out to them.

### Interpretation

This study reinforces existing evidence that HCPs struggled to deliver high-quality care under the pandemic conditions.^14 23^ Due to the rapid and unprecedented nature of the pandemic, those in leadership roles dictated changes to service provision without considering their applicability to clinical settings.^11^ HCPs were frustrated by this level of bureaucracy, which challenged their clinical and ethical judgements.^24^ HCPs felt the dissemination of guidelines was authoritarian rather than women-centred, resulting in impersonal care experiences for service users.^23^ This lack of autonomy perpetuated low morale within the workforce.^25^ Maintaining HCPs’ emotional engagement with work is crucial to enhancing commitment to the job and has shown to be a motivator to carry out high-quality care; thus, future policy must prioritise emotional resilience, even in the context of a health system shock.^26^

Many HCPs recounted going ‘above and beyond’—continuing home visits, creating online information resources and putting their personal safety at risk to counter women’s fear and isolation during the pandemic. Moreover, due to the scaling back of services and isolation from usual support networks, women relied more heavily on HCPs for support.^27^ Ensuring that policy does not trade off the well-being of service users and providers requires continued evaluation, and the need to provide a safe working environment, well-being support and conditions in which high-quality, safe care can be delivered should continue to be a priority.

Additionally, our findings dispute studies which have shown that the pandemic ensued camaraderie among staff, creating a positive working environment.^23^ We found that the pandemic conditions further fragmented the workforce, with the redeployment of HCPs creating workplace dissonance and conflict—findings which align with research identifying redeployment as a risk factor for poorer psychological well-being.^28^ This disruption to staff morale was further highlighted when HCPs reported a lack of communication and information sharing among individual Trusts, contributing to existing reports of the heterogeneity within service reconfiguration across the UK.^29 30^

Trust between HCPs and service users was significantly altered during the pandemic. Participants consistently emphasised the importance of face-to-face communication, relational care, and autonomy in fostering a trusting relationship. The disruption to the organisation of services diminished women’s trust and reliance on maternity services, negatively affecting their care experience and engagement.^31^ Women and partners shared this perspective, reporting communication, HCPs’ attention to concerns and relational care were key issues when receiving maternity care during the pandemic.^6^ The pandemic threatened the patient-provider relationship; the strength of which is crucial in promoting access and utilisation of maternity services.^32^

Additionally, the discrepancy in quality of care among marginalised groups was a significant concern in this study. Participants frequently highlighted the insufficient provision of interpreters during virtual and in-person consultations—exacerbating language barriers known to obstruct access to quality care.^33 34^ Policymakers also highlighted the lack of racial diversity within leadership roles reflecting the underrepresentation of marginalised groups within our policymaking. This aligns with the disappointing reports that communities which have been most affected by the pandemic are those with a longstanding history of poor maternal healthcare.^35^ Community engagement, which was vastly scaled back during the pandemic, has been shown to establish trust between HCPs and patients, especially among marginalised groups.^36^ Therefore, a movement towards community care may purposefully narrow inequalities among marginalised groups.

### Strengths and limitations

This study benefits from rigorous qualitative analysis and adds robust data to the RESILIENT care-seeking and care experience research by enhancing our knowledge of HCPs and policymakers’ perspectives on care delivery and their reflections on the future of maternity services. Where studies have previously focused on individual factors, the construct of Candidacy revealed that care-seeking and care experience is shaped by the intersecting individual-level, health service-level, and joint-level factors. Given the breadth of literature analysing the significant disruption the pandemic posed towards women and their partners, we focus on providing significant data on HCPs and policymakers’ experience of providing care and the longstanding impact the pandemic has had on our service providers. This study makes an important contribution to the existing literature base by incorporating the voices and experiences of HCPs and policymakers, directly impacted by organisation processes and changes in maternity care. Although policymakers have an important role, their perspectives are not often collected in qualitative research due to difficulties in recruitment and scheduling interviews. A strength of the study is the diversity of participants from the four nations of the UK, showcasing the differences in national response.

The data collection period spanned September 2022–February 2023, reflections collected as part of this project encapsulate the entire pandemic period; however, this could have led to recall bias in experiences from the early stages of the pandemic in 2020. The data provided by these participants may not be generalisable to all HCPs and policymakers in the UK. Future research may focus on the perspectives of HCPs and policymakers who reflect a more ethnically diverse population.

### Future policy and practice

The novel coronavirus severely strained maternity services, resulting in poorer engagement and care experiences from service users and providers.^37^ Staff attrition rate within maternity care remains among the highest in the NHS due to low morale, workplace conflict, and poor social and mental well-being.^8^ Postpandemic, 36% of maternity staff reported experiencing burnout, while women consistently reported feelings of isolation and insufficient support.^8 38^ These findings highlight the necessity to take a collaborative (women and HCP-centred) approach to rebuilding a stronger maternity service. The forthcoming 2026 NHS 10-year service delivery plan offers an opportunity to embed the perspective of HCPs and policymakers into reforms, especially under the direction of our newly elected government.^39^ Rather than reverting to a prepandemic model already strained under existing pressures, a forward-facing strategy should prioritise adaptation and innovation.^40^

Our findings support policy directions outlined in the National Institute for Health and Care Research (NIHR) policy brief ‘Advancing co-production in local maternity services’ by highlighting the necessity of centring HCPs' values in service delivery and design.^37^ Consistent, evidence-based guidance and psychological support are essential to sustain workforce well-being and ensure high-quality care.^28^ Furthermore, future healthcare research must intentionally address the unique barriers experienced by marginalised groups through targeted investment in community care and an understanding of social determinants.^36^ This study offers critical insights to inform policy aimed at fostering equity and resilience in maternity care.

## Conclusions

This study affirms the negative indirect effects the pandemic has had on numerous stakeholders, including HCPs, policymakers, women, partners and significantly, marginalised groups. This study has demonstrated that in order to build a resilient maternity system, one that can cope with future health system shocks, policy must prioritise marginalised groups, patient-centredness and the well-being of HCPs. In order to achieve this, we must ensure that strategies to stimulate staff well-being and a working environment which yields pride and satisfaction in work are prioritised. The rapid implementation and standardisation of services do not work for all, therefore, service provision needs to be adapted for marginalised and at-risk groups. Moreover, equal practice among Trusts, including resources, innovations and service provision, is crucial for maintaining equity and equality within our health system. On a local level, we need to place greater value on our community care by ensuring that they are sufficiently staffed and resourced. Within this collaborative approach to improving services, change is possible.

### Patient involvement

The RESILIENT Study benefitted from a Patient and Public Involvement and Engagement Advisory Group (PPIE-AG; n=15), and a Technical Advisory Group (TAG; n=19), both UK-wide, who periodically reviewed data analysis and interpretation. A named PPIE member was involved in validating findings and reviewing this manuscript.

## Supplementary material

10.1136/bmjph-2025-004361 online supplemental file 1

## Data Availability

Data are available on reasonable request.
