# Supplementary material for: Perceptions of maternity care-seeking and care-giving experiences during a health-system shock: a qualitative study with healthcare professionals and policymakers in the UK, with a focus on care for marginalised groups
Source: BMJ Public Health. 2026 Jun 11;4(2):e004361. doi: 10.1136/bmjph-2025-004361 (PMC13264897; doi:10.1136/bmjph-2025-004361)
Supplement: online supplemental file 1 [file bmjph-4-2-s001.pdf]

Post-Pandemic Planning: Interview Schedule- HCPs

Thank you for taking the time to be interviewed in relation to this Post-Pandemic Planning (or what we decide on the final name) project. You are one of the HCPs who have been delivering maternity care during the pandemic- thank you!

Let me tell you a short summary of what we are doing. The RESILIENT project is led by KCL and we are investigating how to plan for the future in maternity services in the UK. We are speaking with women, partners, policymakers and HCPs like yourself to really understand how delivering care was during the pandemic.

I'd like to ask you a few questions to understand the state of maternity services currently, and how you think we can move forward to provide the best maternity care to women. While this will require some reflection of experiences over the time since the pandemic began in Feb 2020, we want to ensure that such reflections are brief, and the focus is on how we move forward in the best way possible.

The interview will be structured in six parts: (1) Current experience, (2) Information sharing, (3) Virtual care, (4) Vaccine hesitancy. (5) Ethics, and (6) Looking forward

We are interested in the full range of your experiences- there are no right or wrong answers, and you will not be judged based on what you say.

The interview will take approximately 30 mins–1hr.

We can provide you with the transcript, should you wish.

Should you feel uncomfortable at any time and wish to stop the interview, or take a break, please tell me.

Do you have any questions before we begin? If not, I shall now start to record.

Just for the sake of recording you are participant number XXX

Section 1: Current Experience

1. **Can you tell me about your day-to-day role and workload <u>currently</u>?**
    a. How does this differ from during the height of the pandemic i.e., during the first wave?
    b. How does it differ to your role prior to the pandemic?

    Probes:

    - Day to day/Logistical (remote working/stepping into other roles/shift patterns/staff shortages)
    - Did you receive support in terms of IT/training/upskilling during this transition?
    - Ability to provide necessary care?
    - Team dynamics? Management?
    - emotional and mental well-being? (Explore- Negative emotions, such as despair, low morale, feeling under-appreciated, anger, helplessness, financial worries)

2. **What were your experiences of raising concerns during the reconfiguration process?**

Probes:

- Did you feel heard? Were you able to suggest making changes and were these taken seriously?

3. **What are some of the biggest challenges for you in your workplace right now?**

Section 2: Information Sharing

1. **Where do you go for your *own* information about COVID-19 infection and vaccination in pregnancy/postpartum?**
    a. How does this differ from the information you use to inform your own practice?
2. **In what format do you like to have your information presented?**
3. **What information do you share with your patients? Or where do you direct them to for information?**

Probes:

- Detailed guidelines
- Quick reference guide/executive summary
- Infographic that can also be shared with patients?

Section 3: Virtual Care & self-monitoring

My next set of questions will address virtual care and women's self-monitoring of particular health problems.

1. **Are you *currently* providing any virtual maternity services yourself?**

Probes:

- Telephone consultations/videoconferences?

2. **Are you *currently* facilitating any self-monitoring in your practice?**
3. **Can you describe what is working well (or not) in provision of virtual care? in self-monitoring?**

Probes:

- For telephone consultations/videoconferences, were there any technological challenges for you or the women? Was digital poverty a problem?
- How was it when the woman's/family's first language wasn't English? How did you manage this? Was it successful?
- Any fears or concerns about the effectiveness or safety of virtual care (vs. face-to-face care) for any particular types of patients (e.g. those suffering from domestic violence)?

4. **How do these activities affect your workload?**
5. **How has your provision of providing virtual vs. face-to-face patient care changed over the course of the pandemic?**
6. **How do you think this has impacted on women's experiences of the care they have received?**

Probes:

- Continuity of care, newborn care, outpatient visits, tertiary services & surgery?

- Any fears or concerns about the effectiveness or safety of virtual care (vs. face-to-face care) or self-monitoring for any particular types of patients (e.g. those suffering from domestic violence)?

Section 4: Vaccine Hesitancy

I'd like to now talk about the COVID-19 vaccine.

1. **What are your views about COVID-19 VACCINATION for women who are planning pregnancy, pregnant, or postpartum?**
2. **What are your views on mandatory vaccination for maternity health care providers? Do you think that this would be justified? Please explain.**

   Probes:

   - On what are these views based? (explore: RCOG? RCM? UK government? Other?)
   - Your views on public messaging (initially non-committal and then reassuring)?
   - Was information available easily? Was it clear? Did it balance known risks of COVID-19 with unknown, theoretical risks of vaccination?
   - Do you have any ongoing doubts?
   - Is there anything you would have liked to see but couldn't find?

Section 5: Ethical Framework

I would now like to ask you to reflect on how care was provided for pregnant and postnatal women in the UK over the course of the pandemic.

1. **What are your thoughts on the care women received.**
2. **What are your thoughts on how HCPs were treated?**
3. **What do you think morally and ethically?**
4. **What changes were/were not justified?**

Probes:

- Explore Q2/3 based on response to Q1- specific ethical opinion on changes; how things changed in height on pandemic vs. later

Section 6: Final Reflections & Looking forward

My final questions are going to ask you to reflect on your personal experiences of delivering care in the pandemic and how you might envisage the future

1. **What has been your major point of learning from the pandemic?**
2. **How do you imagine our best future providing maternity services?**
   i. for yourself as an individual?
   ii. for the healthcare community?
   iii. for women, their families, and their babies?
3. **In your opinion, how do you believe maternity services should be delivered in a future pandemic or other health crisis?**

   Probes:

   - Amend the service
   - 'Hybrid' of service provision practices for before and during the pandemic?
   - Are there practices that will/should go back to normal? Why?

- Are there changes that will/should be stay? Why?
- Do you have any *new* ideas for positive change?
- Agree with vaccination of pregnant/postpartum women outside maternity services?
- Do you believe these opinions are similar to the women in your care?

4. Do you have any other reflections that you feel would be important for us to hear as we imagine our 'best future' for the care of mothers and babies?

Questions to include in online consent form sent prior to interview:

1. At which NHS Trust do you work?
1. What is your current role and how many years have you worked as a qualified [title]?
2. What is your age?
3. How would you describe your ethnicity?
4. *What is your gender?*
    a. *Male/female/non-binary/other/prefer not to say*
5. *Is this the same as what you were assigned at birth?*
    a. *Yes/no/prefer not to say*
6. *What is your sexual orientation?*
    a. *Lesbian/gay/bisexual/pansexual/heterosexual/asexual/other/prefer not to say*
7. Were you personally in a high-risk group that required shielding/self-isolation, or was anyone in your immediate household or family in this group? How did this impact you in your role as a health care-provider?
8. Have you at any point contracted COVID? If so, were you hospitalised?
9. Have you been vaccinated against COVID? If yes, have you or do you plan to have a booster? If you have not been vaccinated, do you plan to be vaccinated? [add prefer not to say option]

Post-Pandemic Planning: Interview themes- Policy Makers

Thank you for taking the time to be interviewed in relation to the RESILIENT project, as someone who was involved in policy-making in maternity care during the COVID-19 pandemic.

I'd like to share with you a short summary of what we are doing in this study.

The RESILIENT project is led by King's College London. We are investigating how to plan for the future maternity services in the UK. We are speaking with women, partners, HCPs and policy-makers like yourself, to really understand your experience in creating maternity care policy.

[Take consent and go through demographic questions]

I'd like to ask you a few questions to understand the state of maternity services currently, and how you think we can move forward to provide the best maternity care to women. While this will require some reflection of experiences over the time since the pandemic began in Feb 2020, we want to ensure that such reflections are brief, and the focus is on how we move forward in the best way possible.

A few additional details that you should know are:

- The interview will be structured in six parts: (1) Current experience, (2) Information sharing, (3) Virtual care, (4) Vaccine hesitancy. (5) Ethics, and (6) Looking forward.
- We are interested in the full range of your experiences. There are no right or wrong answers, and you will not be judged based on what you say.
- The interview will take approximately 30 to 60 mins.
- We can provide you with the transcript of our discussion, should you wish.
- Should you feel uncomfortable at any time and wish to stop the interview, or take a break, please tell me.

Do you have any questions before we begin? If not, I shall now start to record.

Just for the sake of recording you are participant number XXX

1. What was your role in developing policy or service re-configurations within maternity care during the COVID-19 pandemic?

2. **Current experience-** reflections on personal experiences of managing care or developing policy in the pandemic.
   - Can you tell me about any personal reflections on your experiences of developing and delivering maternity care policy during the pandemic?
   - What were the main challenges? What do you think helped or hindered this? How did you work around these challenges?
   - Knowing everything we do now about how the pandemic developed, what would you do differently?

- Is there something you would have like to have done, but couldn’t because of resource limitations or some other reason?

3. **Information sharing-** explore issues surrounding the accessibility of information and guidance about the pandemic, safety guidelines, and vaccinations.
   - What are your thoughts on how information about the pandemic (safety guidance, vaccinations etc.) was shared and communicated to the public during the pandemic?
   - Do you think the information available to the average pregnant person was sufficiently comprehensive? What could have been added?
   - What format of information do you think worked best?
   - What went well? What could have gone better?
   - What information should be added/supplemented in a future pandemic?

4. **Virtual care and self-monitoring**- questions surrounding virtual care and women’s self-monitoring of particular health problems. These will explore what worked well with virtual care, what could be improved, and problems with implementation.
   - Thinking about the need for some of women’s care to be provided virtually during the pandemic, what do you think went well?
   - Do you have any particular reflections on women’s self-monitoring of particular health conditions during pregnancy (e.g., blood pressure? diabetes?)
   - Looking back, what could be improved? Is there anything you would do differently?
   - Specific problems relating to implementation of virtual care?
   - Specific problems related to implementation of self-monitoring?
   - How do you think service reconfiguration and provision of virtual care affected HCPs (workload) and service users (safety) and costs (to the health system and out-of-pocket for service users)?
   - How should care be provided in a future pandemic? How to mitigate any issues *(discussed above*)?

5. **Vaccine uptake and policy** – reflect on views surrounding the COVID-19 vaccine for pregnant women, mandatory vaccination for healthcare providers, and availability of information.

- (if not covered in Q2) What are your views o**n** the COVID-19 vaccination programme for women in the UK, and how information about the risks and benefits of the vaccine were communicated?
- How do you feel about recommendations for mandatory vaccination programmes for health care professionals? Have your views on this changed throughout the pandemic?
- Is there anything about how vaccines (information on safety/risks/benefits/roll out) were presented to pregnant women that you would change in a future pandemic?

6. **Ethical framework**- reflect on how care was provided for pregnant and postnatal women in the UK over the course of the pandemic, from an ethical and moral perspective.
    - Reflecting on the care that was provided in the UK over the course of the pandemic, did you have any concerns from an ethical or moral perspective?
    - What changes need to go and which need to be implemented again in a future pandemic?

7. **Moving forward**- asking participants to envisage the future and how to move forward to best prepare for a future pandemic or health service crisis.
    - Looking to the future, what are your thoughts on how we could best prepare maternity services for a future pandemic or health service crisis?
        - In a future with no restrictions- what would you implement?
        - What changes can we make now to have the biggest impact for a future pandemic?
    - If there is one thing you could put in place now to prepare for similar situations in the future, what would it be?
    - What has been your biggest learning from this experience of creating maternity care policy during a global pandemic?
    - Any other reflections to share?
